# Occult Bacteremia Caused by Enterococcus-Associated Pyuria in an Elderly Man

**DOI:** 10.7759/cureus.45582

**Published:** 2023-09-20

**Authors:** Masato Ishii, Taichi Fujimori, Chiaki Sano, Ryuichi Ohta

**Affiliations:** 1 Family Medicine, International University of Health and Welfare Graduate School of Health Sciences, Tokyo, JPN; 2 Family Medicine, Unnan City Hospital, Unnan, JPN; 3 Community Medicine Management, Shimane University Faculty of Medicine, Izumo, JPN; 4 Communiy Care, Unnan City Hospital, Unnan, JPN

**Keywords:** elderly patients, enterococcus faecalis (e. faecalis), japan, general medicine, family medicine, gram-positive cocci, gram staining, pyelonephritis, bloodstream infection, enterococcus

## Abstract

We report a case of *Enterococcus*-associated pyelonephritis in a 74-year-old Parkinson's patient. He showed constipation, a mild fever, and altered consciousness. Blood cultures revealed Gram-positive cocci (GPC), prompting vancomycin treatment. Urinary Gram staining confirmed pyelonephritis, underscoring its diagnostic utility in elderly patients with vague symptoms. *Enterococcus faecalis* infections can be insidious, with the potential for organ abscesses and persistent fever. Due to nuanced presentations of Gram-positive infections versus Gram-negative ones, diagnosis can be delayed, risking sepsis. Gram-staining urine is vital, especially in older patients, as untreated Gram-positive bacteremia elevates mortality. Given our aging population and their comorbidities, Gram staining's role in quick antibiotic administration is crucial. Hence, its integration into community hospitals is advocated. This case emphasizes early detection and treatment of GPC infections in the elderly and endorses Gram staining for prompt diagnosis of *Enterococcus*-associated pyelonephritis.

## Introduction

Gram-positive cocci (GPC) account for 20% of the causative bacteria in complicated tract infections. *Enterococcus *is the most common GPC bacterium that causes complicated tract infections [1]. *Enterococcus spp.* include *Enterococcus faecalis* and *Enterococcus faecium*. *Enterococcus faecalis* infections are approximately five times more common than *E. faecium* infections. Most *E. faecium* infections are ampicillin-resistant and require treatment with vancomycin, whereas *E. faecalis* infections are rarely ampicillin-resistant. In a cohort of 659 elderly patients hospitalized with complicated urinary tract infections, severe sepsis or septic shock occurred in 50.5% of the patients, and bacteremia occurred in 26% [2]. Gram-negative rods (GNR) and leukocytes in the urine may disappear after antibiotic exposure, and *Enterococci *may be detected as a decrease in bacterial turnover. However, this was not the case in the present study. According to data from 55 immunosuppressed patients, the in-hospital mortality rate due to *Enterococcus *infections other than endocarditis is 43% [3]. Another study reported a mortality rate of 29% [4]. Another study of 205 patients found that patients with enterococcal bacteremia and comorbidities had a seven-day mortality rate of 13% and a 30-day mortality rate of 25% [5]. The mortality rates of GNR and GPC patients who received adequate antimicrobial therapy were 12.8% and 14.8%, respectively, suggesting that enterococcal infections are commonly associated with severe diseases. Enterococcal infections develop in immunosuppressed patients due to the administration of antibiotics or corticosteroids and present with endocarditis, meningitis, osteomyelitis, sepsis, and urinary tract infections [6]. However, the clinical courses of pyelonephritis and bacteremia caused by GPC have not been widely reported.

We encountered a case of *Enterococcus* bacteremia in a patient with a relatively slow, progressive deterioration of consciousness and a low-grade fever. We used this case to illustrate the course of pyelonephritis and bacteremia caused by GPC and examine the diagnosis and treatment strategies.

## Case presentation

A 74-year-old man presented to the emergency department with the chief complaint of constipation. Three years before visiting the hospital, the patient's cognitive symptoms had gradually declined, increasing the burden of nursing care for his wife. Two weeks before admission, the patient presented with constipation. Although the abdominal symptoms were not severe then, constipation persisted for one week, and the possibility of intestinal obstruction was considered. The patient reported no other symptoms. He had a history of osteoporosis, prostatic hyperplasia, prostate cancer, Parkinson's disease, and a Th11 thoracic compression fracture. The patient was administered rebamipide (100 mg/day), levodopa/carbidopa combination (300 mg/day), zonisamide (25 mg/day), mosapride citrate (15 mg/day), elobixibat (5 mg/day), and magnesium oxide (330 mg/day).

The patient’s vital signs on admission were: blood pressure of 119/72 mmHg; heart rate of 99 beats/min; body temperature of 36.4°C; respiratory rate of 16 breaths/min; and partial pressure of oxygen (SpO2) of 98%. His height was 168 cm and his weight was 48 kg (down from 54.2 kg one year earlier); therefore, his body mass index was 17.0 kg/m^2^. The patient was fully conscious, and clinical examination revealed no abnormalities in the cranial nerves or abnormal findings in the head, neck, chest, or abdomen. No obvious costovertebral angle percussion pain was observed; however, tenderness or percussion pain was observed around the thoracic spine. Axillary dryness, a slight decrease in turgor, and tremors in both hands and wrists were observed. Laboratory tests did not show any signs of inflammation (Table 1).

**Table 1:** Initial laboratory data of the patient eGFR: estimated glomerular filtration rate; Na: sodium; K: potassium; Cl: chloride; Ca: calcium; P: phosphorus; Mg: magnesium; CK: creatine kinase; CRP: C-reactive protein; TSH: thyroid-stimulating hormone; T4: thyroxine; SARS-CoV-2: severe acute respiratory syndrome coronavirus 2

Parameter	Level	Reference
Blood		
White blood cells (× 10^3^/μL)	6.5	3.5–9.1
Neutrophils (%)	74.7	44.0–72.0
Lymphocytes (%)	10.4	18.0–59.0
Monocytes (%)	13.9	0.0–12.0
Eosinophils (%)	0.6	0.0–10.0
Basophils (%)	0.4	0.0–3.0
Red blood cells (× 10^6^/μL)	4.55	3.76–5.50
Hemoglobin (g/dL)	14.2	11.3–15.2
Hematocrit (%)	43.5	33.4–44.9
Mean corpuscular volume (fL)	95.6	79.0–100.0
Platelets (× 10^4^/μL)	20.7	13.0–36.9
Total protein (g/dL)	7.0	6.5–8.3
Albumin (g/dL)	4.0	3.8–5.3
Total bilirubin (mg/dL)	0.4	0.2–1.2
Aspartate aminotransferase (IU/L)	30	8–38
Alanine aminotransferase (IU/L)	30	4–43
Alkaline phosphatase (U/L)	131	106–322
γ-Glutamyl transpeptidase (IU/L)	26	<48
Lactate dehydrogenase (mg/dL)	415	121–245
Blood urea nitrogen (mg/dL)	20.7	8–20
Creatinine (mg/dL)	0.80	0.40–1.10
eGFR (mL/min/1.73 m^2^)	72	>60.0
Serum Na (mEq/L)	139	135–150
Serum K (mEq/L)	4.1	3.5–5.3
Serum Cl (mEq/L)	101	98–110
Serum Ca (mg/dL)	9.7	8.8–10.2
Serum P (mg/dL)	3.0	2.7–4.6
Serum Mg (mg/dL)	2.2	1.8–2.3
CK (U/L)	224	56–244
CRP (mg/dL)	0.11	<0.30
TSH (μIU/mL)	0.84	0.35–4.94
Free T4 (ng/dL)	1.1	0.70–1.48
SARS-CoV-2 antigen	Negative	Negative
Urine		
Leukocyte	Negative	Negative
Nitrite	Negative	Negative
Protein	(+1)	Negative
Glucose	Negative	Negative
Urobilinogen	normal	normal
Bilirubin	Negative	Negative
Ketones	Negative	Negative
Blood	Negative	Negative
pH	6.5	
Specific gravity	1.021	

**Figure 1:**
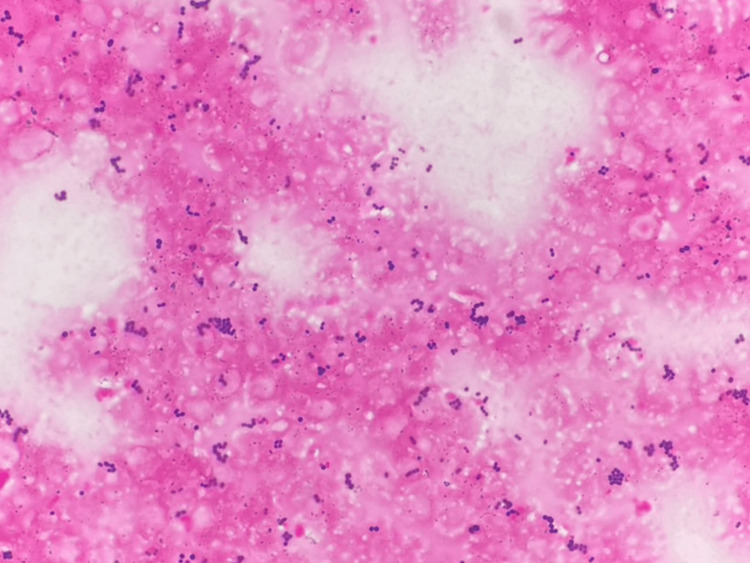
Gram stain of the blood culture showing Gram-positive cocci dispersed throughout the field

On the third day of hospitalization, the patient had a fever of 38°C, and considering the possibility of bacteremia, we performed a blood culture. He had no chills, changes in respiratory rate or blood pressure, or a decrease in consciousness level. The Quick Sequential Organ Failure Assessment (qSOFA) score was one. On the fifth day of hospitalization, GPC was observed in blood culture, as confirmed by Gram staining (Figure 1).

**Figure 2:**
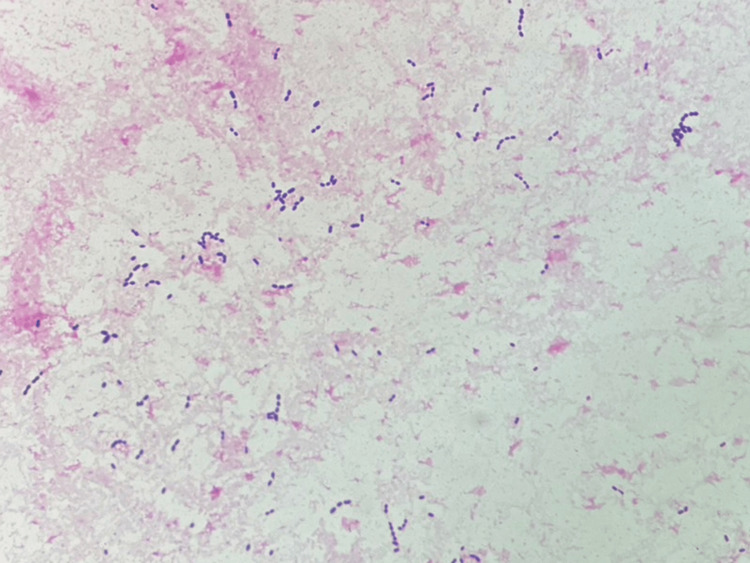
The Gram stain of the urine showing Gram-positive cocci dispersed throughout the field

The mortality rate in elderly patients with sepsis due to GPC infections is high, especially those with comorbidities [5]. The patient had cancer, was undernourished, and had other comorbidities. As the mortality rate has been reported to increase by 7.6% with each delay [7], we initiated empiric antibiotic administration of vancomycin (1 g, 12-hourly), a broad-spectrum antibiotic for GPC targeting *Enterococcus faecium* resistant to ampicillin. On the same day, Gram staining of the urine was performed, and GPC was observed in the chains (Figure 2).

Blood cultures and urine Gram staining suggested GPC bacteremia due to pyelonephritis. On day seven, the patient was de-escalated to ampicillin (1 g, eight-hourly), based on the results of blood and urine cultures of *Enterococcus faecalis* sensitive to ampicillin. A 14-day course of antibiotics was administered; the fever subsided, and the patient's appetite improved. Owing to the lack of improvement in his activities of daily living, the patient was discharged from the hospital on the 28th day.

## Discussion

In this case, during the follow-up of an older man with Parkinson's disease who was hospitalized for a fever of unknown origin, Gram-positive bacteremia was discovered, and the patient was treated for pyelonephritis as soon as possible. Generally, enterococcal pyelonephritis does not progress rapidly unless the patient exhibits marked immunosuppression. We decided to provide treatment based on the discovery of GPC by urinary Gram staining and the patient’s mild disturbance of consciousness, although the patient was stable.

Treatment should be initiated as soon as possible for immunosuppressed patients. Infection with *Enterococcus faecalis* is long-lasting and can lead to the formation of microabscesses in the prostate, intestinal tract, and kidneys [1,2]. Contrary to the rapid progression of Gram-negative bacilli bacteremia and pyelonephritis, GPC infection with *Enterococci *may not reveal apparent abnormalities on physical examination and imaging studies [3,5]. As a result, the infection progresses, and the possibility of causing bacteremia increases.

In this case, diagnosing a mild, persistent, low-grade fever of unknown origin can be challenging. To establish a diagnosis, it is necessary to test for suitable blood cultures and other available specimens. In general medical practice, fever of unknown origin can originate in several organs [3]. The proportion of patients with GPC infections has also increased [1]. Furthermore, owing to an aging society, it is necessary to deal with multi-disease comorbidities, and patient conditions are diversifying [5]. Patients with a combination of fever and indefinite complaints, as in this case, require management by a medical professional specializing in general practice, and future clinical practice and education are expected to improve.

In the medical care of elderly patients with fevers of unknown origin, the principle of fever medical care is the most important; among these, the importance of Gram staining should be emphasized. In this case, the presence of GPC on urinary Gram staining led to the treatment of pyelonephritis. The detection of GPC by urinary Gram staining may be an indication for treatment, even in patients with mild disturbances in consciousness and stable vital signs. Previous reports have shown that sepsis caused by GPC has a high mortality rate [5], and a one-hour delay in the administration of antibiotics increases the mortality rate by 7.6% [7]; therefore, treatment is needed even if the qSOFA scores are low. Previous reports have shown that Gram staining is a helpful test for treating infectious diseases, and using Gram staining may enable optimal treatment.

Furthermore, according to a study comparing antibiotics selected by trainees in the emergency department by point-of-care Gram staining with antibiotics hypothetically selected based on guidelines, carbapenems, and anti-MRSA drugs, only 4.8% of the point-of-care Gram-stained group chose broad-spectrum antibiotics, compared to 44.7% based on guidelines [8]. There was no difference in the effectiveness of antibiotics (point-of-care Gram staining 89.4% vs. guideline 91.8%), and the drug price of point-of-care Gram staining was within 40% of the guideline [8]. Gram staining was effective in suppressing broad-spectrum antibiotics. It may be possible to shorten the timing of antibiotic treatment by selecting the appropriate antibiotic and monitoring the number of bacteria, which may reduce costs. Gram staining has been reported to have a sensitivity of 95.4% and a specificity of 87.2% in the case of complicated pyelonephritis (including prostatitis), which can be caused by many different types of bacteria [9]. Blood and urine cultures take several days; therefore, performing Gram staining makes it possible to select an appropriate antibiotic early, with a high probability, without waiting for the results of antibiotic susceptibility testing.

Gram-positive cocci urinary tract infections are more likely to cause sepsis in older adults [10]. Early Gram staining can shorten the time to diagnosis, enabling the administration of appropriate antibiotics as soon as possible [11]. This is likely to reduce mortality rates. When treating infectious diseases in local hospitals, general physicians who treat patients with infections in multiple organs must understand the usefulness of Gram staining, perform appropriate Gram staining, and promptly administer antimicrobial treatments based on the results [12,13].

## Conclusions

Because enterococcal pyelonephritis rarely progresses rapidly in patients who are not severely immunosuppressed, it is essential to combine blood cultures and Gram staining for early diagnosis and treatment. In addition, if GPC is discovered on urinary Gram staining, antibiotic treatment should be considered even in patients with mild disturbances of consciousness and stable vital signs. Accurate and rapid Gram staining may improve the quality of care.
